# Characterizing the role of adjoining twins at grain boundaries in hexagonal close packed materials

**DOI:** 10.1038/s41598-019-40615-5

**Published:** 2019-03-07

**Authors:** M. Arul Kumar, L. Capolungo, R. J. McCabe, C. N. Tomé

**Affiliations:** Materials Science and Technology Division, Los Alamos National Laboratory, Los Alamos, NM 87545 USA

## Abstract

Hexagonal close packed (HCP) Mg and Zr are being used in transportation and nuclear industries, respectively. The ductility and formability of these materials is significantly limited by the activation of prevalent deformation twinning. Twins in HCP polycrystals usually nucleate at grain boundaries (GBs), propagate into the grain, and they either terminate at opposing GBs (isolated-twins) or transmit into a neighboring grain (adjoining-twin-pairs: ATPs). Because twin interfaces provide a path for crack propagation, twin transmission is relevant to material ductility. This study combines electron backscatter diffraction (EBSD) based statistical analysis of twinning microstructures and crystal plasticity modeling, to characterize twin thickening processes away from and near GBs. Analysis of deformed Mg and Zr microstructures reveals that local twin thicknesses at GBs are statistically larger for ATPs compared to isolated-twins. Further, thicknesses are found to decrease with increasing GB misorientation angle. Full-field Fast-Fourier-Transform micromechanics modeling shows that shear-transformation induced backstress are locally relaxed at GBs for ATPs, but not for isolated-twins. As a consequence, ATPs can thicken locally at GBs and the preferential site for twin thickening shifts from the middle of the twin to common GB.

## Introduction

Deformation twinning is a prevalent plastic deformation mode in hexagonal close packed (HCP) metals due to a scarcity of easy crystallographic slip modes to accommodate arbitrary loading^1,2^. In polycrystals, most twins nucleate at grain boundaries, where favorable stress concentrations and defects are present^3,4^. Under continued straining, the nucleated twins propagate into the grains and usually terminate at opposing grain boundaries. Further, the forward stress induced by the twinning shear can lead to the formation of a twin on the other side of a grain boundary (GB). Sometimes this process continues across several GBs and creates the so-called twin chains or catalytic twins^3,5–10^. In this work, two twins that are connected at a grain boundary are referred to as adjoining twin pairs (ATPs), while twins that terminate at grain boundaries are called isolated twins. Isolated twins and ATPs both affect the mechanical and failure response of metals^11,12^. In particular, ATPs act as preferred sites for instabilities such as void nucleation, cracking, and premature failure^13–17^. For example, Bieler *et al*.^16,17^ and Simkin *et al*.^13^ showed that cracks nucleate at twin-GB junctions due to the local strain heterogeneity developed by the twin interactions with the GB.

Under continued straining, both isolated twins and ATPs thicken. Overall, Electron Back Scatter Diffraction (EBSD) observations show that the thickness of twins vary irregularly as a function of distance from grain boundaries. Indeed, twins either adopt an elliptical shape (i.e. are thicker away from the GB) or are found to be thicker in the neighborhood of GBs^7,18,^^19^. While the mechanisms affecting local thickening are not well understood, it is logical to assume that they are driven by local stress fields. Note that deformation twins are diffusionless and displacive transformation-induced process, which is significantly different from the diffusive thermally activated annealing twins. Thus, the motion of deformation twins differs from the one of annealing twins in that their propagation is not driven by surface tension but by the local stress fields. Recently, a series of studies were performed to understand the stress field in the vicinity of isolated twins using crystal plasticity modeling tools^8,20,21–24^. It was found that the twinning shear transformation is accompanied by stress reversal, i.e. ‘backstress’, within and in the vicinity of twins^18,25^. The backstress depends on the plastic properties and orientation of the neighboring grain that accommodates the twinning shear at the twin tip-GB junction^8,24^. For all possible neighbor orientations, the backstress is high at the twin tip and relatively low at the twin middle^8,18^. This suggests that twins may start to thicken preferably from the middle, not from the twin tip-GB junction^24^. This leads to lenticular shape twins, which is the most commonly observed twin shape in HCP polycrystals^7^,^18,19^. To develop similar understanding of twin thickening process for ATPs, the local stresses associated with ATPs need to be characterized, particularly at a common GB where twins are connected. This will reveal whether the formation of ATPs significantly changes the driving force for the nucleation and propagation of disconnections mediating twin thickening.

Studies on dislocation interactions with GBs performed over several decades have found that the GB properties and local interactions have a strong role on polycrystal deformation behavior and on cracking^26–30^. However, the role of GB nature on ATP formation and further thickening has received less attention. More recently, the relationship between misorientations at GBs and twin transmission or ATP formation has been studied for a series of HCP metals including magnesium and its alloys^3,6–8,10,31–34^, zirconium^8^, titanium and its alloys^9,35^, and rhenium^36^. A common feature from all these studies is that the twin transmission frequency decreases with increasing GB misorientation angle. By combining EBSD based statistical analysis and full-field elasto-visco-plastic Fast Fourier Transform (EVP-FFT) calculations, we found that plastic anisotropy, which quantifies the differences in the critical stress for slip modes, correlates with twin transmission^8^. Twin transmission is more likely for higher plastic anisotropy for the same GB misorientation angle^8,32^. All these works, however, do not explain the local reactions of the ATPs at the GBs.

In this work, we characterize the twin thickening process of ATPs using experimental statistical analysis and crystal plasticity modeling. Detailed twin statistical analysis is performed in deformed HCP magnesium and zirconium. From the statistical analysis we find that ATPs are thicker at GBs than midway through the grain (i.e., twins forming ATPs are not lenticular). The local twin thickness at GBs of ATPs is also found to be systematically larger than that of isolated twins. To rationalize these findings, we employ a full-field micromechanics crystal plasticity modeling tool. Specifically, an Elasto-Visco-Plastic Fast Fourier Transform (EVP-FFT) model that explicitly simulates the reorientation and shear transformation associated with deformation twins^18,24^. This model calculates micro-mechanical fields of Cauchy stress tensor, elastic and plastic strain tensors, and so on, at every material point and allows to calculate the driving forces for further twin expansion. We have performed twinning simulations for both, isolated and transmitted twinning configurations, to understand the differences in the twin thickening driving force. Static simulations show that ATPs relax and reverse the backstress locally and thus favor the twin thickening at GBs. A similar local relaxation does not take place for isolated twins, which inhibits their ability to thicken at GBs.

## Results

### Experimental characterization

**Figure 1:**
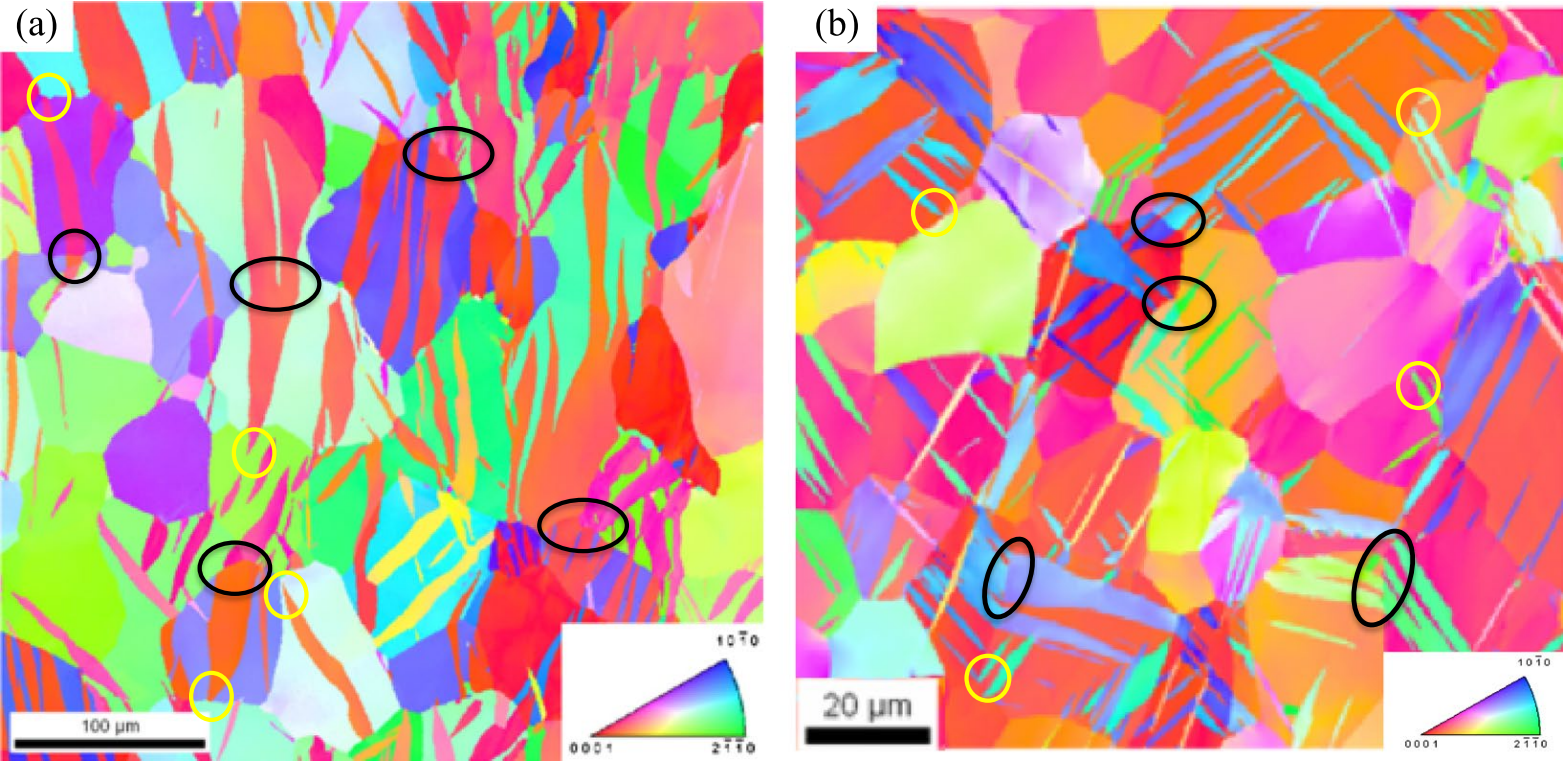
Tensile twinning microstructure in HCP metals. Electron backscatter diffraction (EBSD) image of (**a**) Mg compressed to 3% strain at 273 K and (**b**) Zr compressed to 10% strain at 77 K shows extensive activation of $\{10\bar{1}2\}$ tensile twinning. EBSD images are cleaned using Grain confidence index (CI) standardization cleanup followed by an iterative Neighbor CI correlation with a minimum CI of 0.3. A few isolated (yellow) and ATPs (black) twins are marked.

High-purity polycrystalline Mg and Zr were used for this study^7,19^. Both materials have a strong basal texture resulting from rolling, and hence, similar grain boundary misorientation distributions. To activate $\{10\bar{1}2\}$ tensile twinning, both materials were compressed at 10^−3^/s along an in-plane direction. To activate a sufficient number of twins in many grains, Mg was compressed to 3% at room temperature, whereas Zr was compressed to 10% at 77 K. Deformed microstructures for both materials were mapped using EBSD and the collected maps were analyzed using an automated twin analysis software^7,8,19,37,38^. Refer methods section for more details about the microstructural mapping and statistical analysis. Representative EBSD images of compressed Mg and Zr are shown in Fig. 1 clearly showing extensive activation of tensile twins. The number of grains, twins and grain boundaries investigated in this work respectively are: 2339, 8550 and 23321 for Mg, and 639, 1065 and 4839 for Zr. Here the grain boundaries do not include twin interfaces. For the Mg, 11615 GBs out of 23321 GBs have at least one impinging twin (49.8%) and 3407 grain boundaries have ATPs (14.6%). Similarly, in Zr, 2275 GBs out of 4839 GBs have at least one twin (47.0%) and 1020 grain boundaries have ATPs (21.1%).

From the geometric viewpoint, grain boundaries have 5 degrees of freedom. For simplicity, however, here we adopt the usual practice of representing misorientation by a single scalar misorientation angle. The misorientation angle θ across a GB adjoining grains 1 and 2 is,

1 $$\theta =min\{{\cos }^{-1}(\frac{tr({R}_{1}{R}_{2}^{T})-1}{2})\}$$

Where R1 and R2 are the rotation matrices that transform the crystal axes of grains 1 and 2 into sample frame, respectively. Here the crystal symmetry is considered in the rotation matrices, and so the calculated angle θ is the minimum misorientation angle between all possible equivalent symmetries.

**Figure 2:**
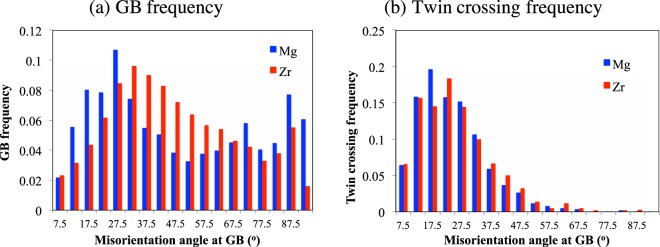
Statistical analysis of adjoining twin pairs in HCP metals. Statistical distribution of (**a**) grain boundary frequency and (**b**) twin crossing frequency as a function of GB misorientation angle for pure Mg and Zr. The twin crossing after ~50° GB misorientation is statistically negligible.

Figure 2(a) shows the frequency of GB misorientation angles for both Mg and Zr. It confirms that in the studied materials there are sufficient grain boundaries for every misorientation angle bin to be statistically representative. Note that while the initial material has a strong basal texture, GB misorientations span the range of all possible misorientations. In Fig. 2(b) the distribution of twin crossing (or ATPs) is shown and reveals that twin crossing is statistically significant for low misorientation angle GBs and it is almost negligible for GBs with misorientation angle greater than ~50°. A Similar observation has been already reported by Kumar *et al*.^8^. As such, for the rest of the study, only the GBs whose misorientation angle is less than 50° is considered.

To identify the preferential site for twin thickening and to quantify the local twin reactions at GBs, the twin thickness at and far away from the GBs for both the isolated twins and ATPs measured. The twin thickness midway through the grain is obtained by fitting an ellipse to the twins and identifying the thickness as the dimension of the minor axis of the fitted ellipse. The actual twin plane is generally inclined to the scanning surface, so the true twin thickness is estimated by multiplying the projected twin thickness by the cosine of the angle between the twinning plane, K1, and scanning surface normal^7,19^. For the sake of simplicity, TTM denotes the twin thickness midway through the grain. The evolution of TTM as a function of grain size and grain orientation is shown in Figs 12 and 10 of Beyerlein *et al*.^7^ for Mg, and Figs 12 and 6 of Capolungo *et al*.^19^ for Zr. The net average TTM thickness is 3.01 μm and 0.30 μm for Mg and Zr, respectively. Note that the twin morphology in Mg is usually not lenticular and so fitting of an ellipse may not be appropriate. The fact that twins in Mg are thick compared to Zr, was shown to be a consequence of their elastic and plastic anisotropy^24^. The high anisotropy in the critical strength of slip modes in Zr leads to higher back stress and more resistance for twin growth compared to Mg for similar GB misorientation and, as a result, Zr twins cannot thicken as much as Mg ones^24^.

**Figure 3:**
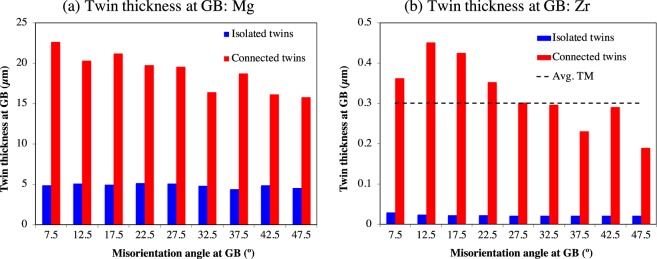
Statistical analysis of twin thickness at GBs for HCP Mg and Zr. The distribution of twin thickness at grain boundaries (TTGB) as a function of GB misorientation angle for both isolated twins and ATPs in (**a**) Mg and (**b**) Zr. Twin thickness is significantly higher for ATPs compared to isolated twins. Average twin thickness midway through the grain, TTM, for Zr is also shown for comparison. Because Mg twins are not lenticular, calculating TTM by fitting an ellipse is not considered here.

The twin thickness at GBs is the length of the twin-GB junction along that grain boundary, which is denoted by TTGB. In this work, the twin thicknesses at GBs for both isolated twins and ATPs are measured. This is shown in Fig. 3 as a function of GB misorientation angle. Qualitatively, the twin thickness distribution is similar for both Mg and Zr, but not the absolute values. Similar to TTM, the TTGB is also significantly higher for Mg compared to Zr. From the statistical distribution shown in Fig. 3, the following observations are derived: (i) TTGB is always high in ATPs compared to isolated twins for all misorientation angles; (ii) TTGB decreases with increasing misorientation angle for ATPs, while this trend is not seen for isolated twins; (iii) for isolated twins, TTM is comparable or higher compared to TTGB, (iv) for ATPs, TTM is low compared to TTGB for both Mg and Zr. From these observations we can conclude that the preferential site for twin thickening is the twin middle for isolated twins, and the common GB when twins are connected as ATPs. The underlying mechanism for such a shift in the twin thickening sites is discussed in the following section.

### Crystal plasticity modeling

**Figure 4:**
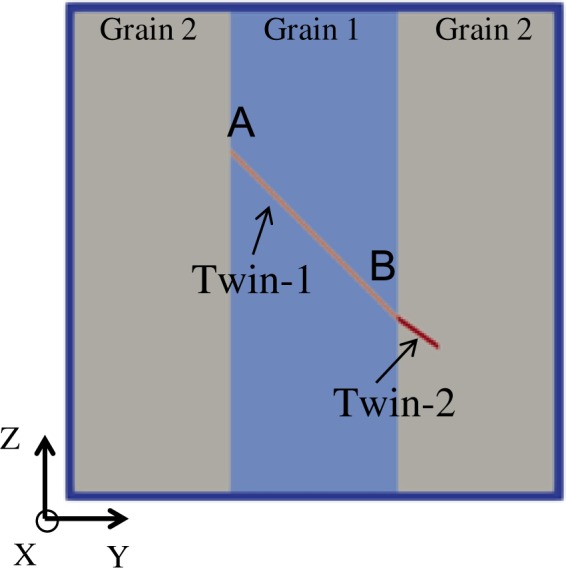
Micromechanics model for twin stress calculation. Tri-crystal setup with ATPs that used for the local stress calculations using EVP-FFT model. In the calculation, first twin-1 is introduced in the central grain (grain-1) and later twin-2 in the neighboring grain (grain-2) is introduced to from ATPs.

To investigate the role of twin-GB interactions on the observed twin thickness distribution, a full-field elasto-visco-plastic Fast Fourier Transform (EVP-FFT) model that accounts for anisotropic elasticity, crystal plasticity, and explicit discrete twin lamellae formation within the polycrystals^18,24^ is employed. The model calculates the spatially resolved stress fields associated with twinning transformation in HCP polycrystals. Refer methods section for more details about the model. In this work, both the isolated and ATPs are simulated using the EVP-FFT model to study the local stresses that drive twin thickening at GBs. Figure 4 shows the model setup of a 3D tri-crystal, which consists of a central grain surrounded by two equal-sized neighboring grains with the same crystal orientation. The c-axis of the central grain is oriented along the z-direction, which corresponds to the Euler angles of (0°,0°,0°) in Bunge convention. Different neighboring grain orientations are chosen to study the effect of neighbors on twin local stresses. A buffer layer with almost random grain orientation distribution surrounds the tri-crystal. The entire unit cell is discretized into 3 × 750 × 750 voxels with the buffer layer thickness of 10 voxels.

The details of the static twinning calculation are as follows. The model tri-crystal is compressed along the Y-direction and thus it favors the activation of a (01-12)[0-111] tensile twin in the central grain. For simplicity, we have chosen neighboring grain orientations that will only form tilt GBs, such that the imposed compression along Y-direction also favors activation of the same tensile twin in the neighboring grain. A set of voxels is pre-selected for twin activation in the central and in the neighboring grain as shown in Fig. 4. The simulations are performed in a sequence: first the twin free tri-crystal is subjected to compression along the Y-direction; then the tensile twin in the central grain (grain 1) is introduced; later the tensile twin in the neighboring grain is introduced. The formation of twins in the model refers to twin reorientation of the pre-selected voxels and explicit accommodation of the associated twinning shear. The deformation in the calculation is accommodated by a combination of anisotropic elasticity and crystallographic slip induced plasticity. The anisotropic elastic constants (in GPa) for Mg at room temperature are: C11 = 58.58, C12 = 25.02, C13 = 20.79, C33 = 61.11 and C44 = 16.58^39,40^, and for Zr at 76 K are: C11 = 154.20, C12 = 67.80, C13 = 64.80, C33 = 171.60 and C44 = 35.80^40,41^. Plastic deformation is accommodated by basal <a>, prismatic <a>, and pyramidal-II <c+a> slip systems for both HCP Mg and Zr metals. The critical resolved shear stress (CRSS) for basal, prismatic and pyramidal slip systems (in MPa) are 3.3, 35.7 and 86.2, respectively for Mg at room temperature^42^; 700.0, 20.0 and 160.0, respectively for Zr at 77 K^43^.

**Figure 5:**
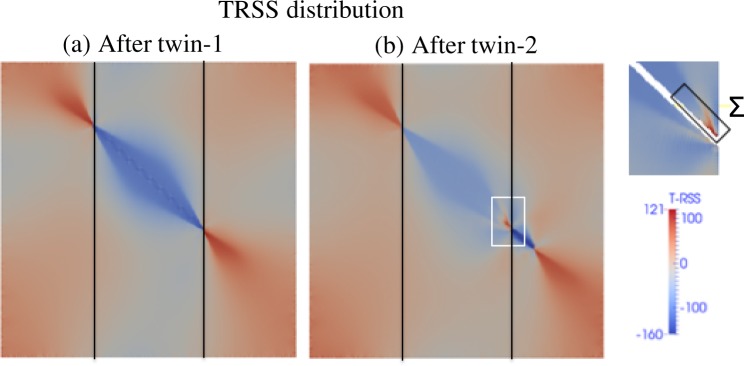
Distribution of stresses within and in the vicinity of twins. The distribution of twin-plane resolved shear stress with respect to twin -1 (TRSS-1) (**a**) after twin-1 and (**b**) after twin-2 formation at a fixed strain. The crystal orientation of grain 1 and grain 2 are (0°, 0°, 0°) and (0°, 10°, 0°), respectively, in Bunge convention, and it corresponds to 10° tilt GB. The formation of twin-2 relaxes the back stress of twin-1 locally at twin tip and it may facilitate local twin thickening at GB.

Using the EVP-FFT model^18,24^ we have calculated the micromechanical fields such as the elastic and plastic strain tensors and Cauchy stress tensor at every FFT voxel for both Mg and Zr during the proposed sequence of twin domain insertions. The resolved shear stress on the twin plane along the twin direction (TRSS) is used to compute the driving force acting on the twin domain. The TRSS can be calculated with respect to twin-1 and twin-2. In this work we are interested in the thickening of twin-1 (i.e. the first twin inserted in the microstructure) at a common GB and so we only present the TRSS with respect to twin-1 (TRSS-1). The distribution of TRSS-1 after the insertion of twin-1 in the central grain is shown in Fig. 5(a) for the case of a 10° tilt GB for Zr. Note that before twinning, the stress field is homogenous and positive (~100 MPa) in the central grain. The formation of twin-1, i.e., accommodation of twinning shear, creates a stress reversal in the central grain and so the twin cannot thicken without further loading^23,44^. At the same time, a stress concentration develops in the neighboring grain at the twin tip. It favors the nucleation of a twin in the neighboring grain, which can lead to twin transmission/adjoining twin pair formation. In our previous work we studied the effect of neighboring grain orientation along with elastic and plastic anisotropy on twin transmission^8^. In the present work, after twin-1 we introduce the second twin in the neighboring grain. The distribution of TRSS-1 after twin-2 formation is shown in Fig. 5(b). The backstress in the surroundings of twin-1 is still present, however the activation of twin-2 alters the stress field at the twin-1 and GB junction. The enlarged view of a small region of the central grain that encompasses the twin-1 and twin-2 connection at GB is shown in Fig. 5. The stress reversal observed after twin-1 formation relaxes locally and as a result the TRSS-1 becomes locally positive at the twin-1 and GB junction.

**Figure 6:**
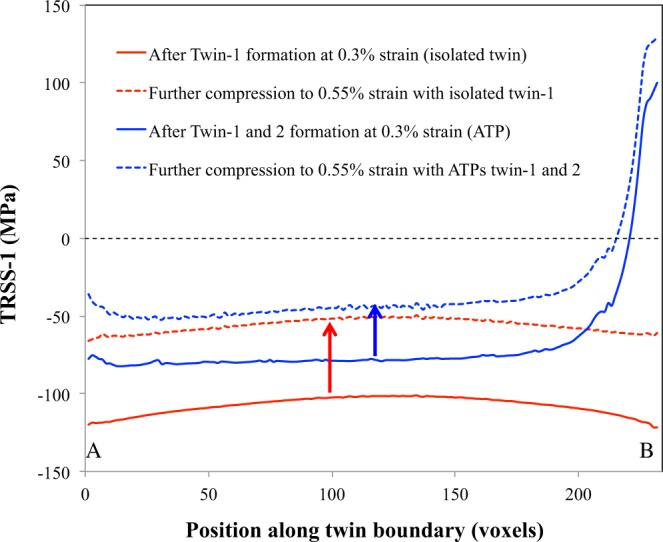
Effect of connected twin on TRSS along twin interface. The TRSS-1 profile along twin-1 interface (from A to B, see Fig. 4) after isolated (twin-1) and ATP (twin-1 and 2) formation and after further compression. The continuous and dashed red lines correspond to the TRSS-1 profiles after the formation of isolated twin at 0.3% strain and after further compression to 0.55% strain with only the isolated twin. Similarly, the continuous and dashed blue lines correspond to the TRSS-1 profiles after the formation of ATP at 0.3% strain and after further compression to 0.55% strain with ATP.

The TRSS-1 profile along the twin-1 top interface from A to B (see Fig. 4) is plotted in Fig. 6. The TRSS-1 profile after formation of the isolated twin-1 (continuous red curve) shows that the stress reversal is higher at the twin tip compared to twin middle. Under further straining, twin thickening may start at the middle of the twin, and not at the tip. To confirm this, we impose further macroscopic compression (0.55% strain) to the tri-crystal with the isolated twin and the corresponding TRSS-1 profile is plotted (dashed red curve). The stress profile does not change much with further loading such that the most preferable site for twin thickening is still the twin middle. This stress profile explains why the TTM is high compared to TTGB for isolated twins.

The TRSS-1 profile after the formation of twin-1 and twin-2 (continuous blue curve) clearly shows the relaxation of twin backstress associated with twin-1 locally at the GB (location B), and as a result TRSS-1 is significantly high and positive. It suggests that after the formation of ATPs, twins prefer to thicken near the common GB where the ATP is formed, not at the twin middle. Similar to the isolated twin case, imposing the same macroscopic compression (0.55% strain) to the tri-crystal with the ATP (twin-1 and 2) causes the TRSS-1 to increase in the positive direction. The corresponding TRSS-1 profile (dashed blue line) confirms that the stress profile will not change drastically with further loading and so twin thickening should be more favorable at the twin-GB junction for ATP and not at the twin middle. This explains the experimentally observed high TTGB compared to TTM for ATPs. The local relaxation of backstresses observed for ATPs is not available for isolated twins. Thus, for the same configuration (same twin type, same parent and neighboring grain orientations) the TTGB for isolated twins will be lower than the ATPs in agreement with the experimental observations shown in Fig. 3.

To quantify the local relaxation of twin back stresses due to the formation of ATPs, we calculate the average TRSS-1 in a small region, Σ (see Fig. 5), of the central grain where twin-1 is terminated at the GB. The calculated average TRSS-1 in region Σ is −85.7 MPa after twin-1 formation and it becomes 92.7 MPa after twin-2 formation. In HCP Mg we also observe a similar local relaxation of twin-1 back stresses as a result of twin-2 formation. The fact that the relaxation is smaller (in relative values) for Mg compared to Zr would explain the statistical result reported in Fig. 3, namely, that the ATP thickness is larger in Zr. As a closure, to study the effect of neighboring grain orientation on the local backstress relaxation we repeated the calculation for two more tilt GBs (5°, 20° and 30° tilt GB) for both Mg and Zr. The average TRSS-1 in the region Σ after twin-1 and twin-2 formation for both Mg and Zr is given in Table 1. The amount of local backstress relaxation decreases with increasing misorientation angle for both Mg and Zr. Particularly, the local back stress relaxation for the 30° misorientation case in Mg is significantly lower than other misorientation cases. It is due to the relative orientation of easy basal slip system of grain-1 with respect to twin-2. For the case of 30° misorientation, the basal slip system of grain-1 is well aligned with twin-2 compared to other cases. Thus, the twin-2 shear is easily accommodated by the basal system of grain-1 and so the twin-1 back stress is not relaxed significantly. Overall this suggests that the local thickening of ATPs at the GB will decrease with increasing misorientation angle, which is also consistent with the experimental observation shown in Fig. 3.

**Table 1:** Average TRSS-1 in region Σ (see Fig. 4) after the formation of isolated twin-1 and ATPs twins-1 and 2 for both Mg and Zr corresponding to different GB misorientation angles.

Misorientation angle at GB	TRSS in region Σ			
	Mg		Zr	
	After twin-1	After twin-2	After twin-1	After twin-2
5°	−50.4	35.5	−85.0	101.8
10°	−49.5	29.1	−85.5	92.7
20°	−41.4	16.9	−86.5	79.2
30°	−37.1	−33.9	−89.1	48.7

### Preferential twin thickening sites

**Figure 7:**
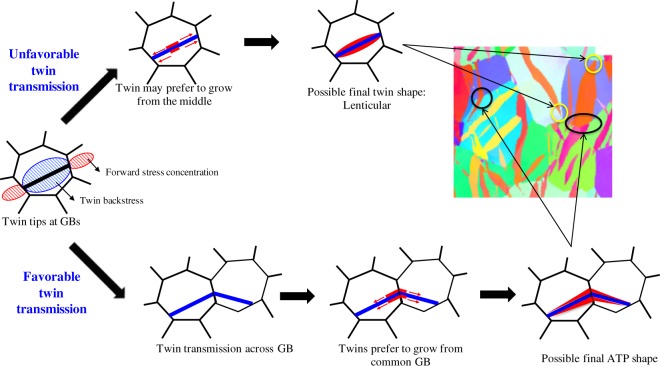
Twin thickening process for isolated and connected twins. Schematic representation of the sequential steps involved in the twin thickening process for an isolated twin and ATPs. Twin tips that either terminate at grain boundaries or that connect another twin in the neighboring grain are marked in yellow and black, respectively.

Twin statistical analysis shows that the twin thickness at GBs is high for ATPs compared to isolated twins. Crystal plasticity modeling reveals that the backstress associated with twinning shear transformation is relaxed locally at a common GB by the formation of twin in the neighboring grain, which favors the local twin thickening at the GB. By combining these results, we conclude that the preferential site for twin thickening will shift from the twin middle to the common GB for ATPs. This is schematically shown in Fig. 7 along with an example from an EBSD map of Zr. The length scale of the present modeling framework is not capable to resolve the atomistic level microstructural variations and its role on twin thickening process. As a consequence, here we only study effect of the intra-granular stresses on local twin thickening process, not the effect from local variations in defect structure and interactions at the twin interface. First, we start with the isolated twin that is terminated at the GBs. Under straining, based on the stress profile shown in the Fig. 6 for isolated twins, we conclude that twin thickening is likely to start at the twin middle and extend outwards along the twin shear direction. For isolated twins this leads to lenticular shapes, which are most commonly observed in experiments^19,44,45^. (see schematic in Fig. 7). In the case of ATPs, and based on the stress profile shown in Fig. 6, we argue that twin thickening will start preferentially at the common GB, not at the middle. The twins will then grow from the GB into the grain leading to a wedge shape rather than a lenticular shape. As a result, the twin thickness will be higher at the common GB, not at the twin middle, which explains the experimental observation reported in Sec. 2 (see schematic in Fig. 7).

In summary, in this work the effect of twin reactions at grain boundaries on thickening of isolated twins and ATPs was studied using experimental characterization and crystal plasticity modeling. A detailed statistical analysis of twins in HCP Mg and Zr reveals that the twin thicknesses at GBs are substantially larger for ATPs compared to isolated twins. In addition, the twin thickness at grain boundaries decreases with increasing misorientation angle, because the crystallographic alignment for transmission decreases. Also, the average thickness at the middle of the twins is high compared to twin thickness at GBs for isolated twins, and vice versa for ATPs. Using full-field crystal-plasticity calculations, we show that the transmitted twins accommodate and relax the twinning shear and so favor local thickening at grain boundaries. Such local twin shear accommodation is not possible for isolated twins and as a result they do no thicken at grain boundaries. Using these results, we conclude that the preferential site for twin thickening is the twin middle for isolated twins and the common GB for ATPs.

For either twin nucleation or thickening, both the interface defects and their interactions with mechanical stresses should be favorable^46–52^. In this work we only study the mechanical stresses and not the microstructural defect interactions. The interaction of dislocations with twin interfaces has been studied using *in-situ* high resolution microscopy and atomistic calculations, but only for isolated twins inside the grain, not at or near GBs^46–52^. Such interactions with the twin interfaces may be sufficient for explaining growth of isolated twins, because they will preferably take place at the grain interior and not at the GB. But for ATPs, the local reactions at GBs need to be explored. To develop a more comprehensive understanding of twin-GB interactions and associated local processes, we plan in the future to tackle a 3D characterization both, at the experimental and the computational level. However, the present work elucidates the local micromechanical processes associated with isolated and ATPs and provides a starting platform for future research.

## Methods

### Statistical characterization

For twin statistical analysis, EBSD scans were performed with a step size of 1.0 and 0.2 microns for Mg and Zr, respectively. To develop useful statistical correlations, a large number of grains and twins need to be analyzed. Our statistical datasets for Mg and Zr are from forty-two distinct 400 μm × 600 μm scans and six distinct 120 μm × 240 μm scans, respectively. The acquired EBSD images were processed using the automated EBSD-twinning analysis software, METIS^37,38^. This software identifies all grain and twin boundaries by combining analysis of neighboring point misorientations with graph theory. The twin recognition module identifies twin domains and their corresponding twinning types by comparing each boundary misorientation, both the axis and angle, with the appropriate twinning relationships. The twin thickness at the middle is estimated by fitting an ellipse to the twins with the projected twin thickness in the analysis section defined as the dimension of the minor axis of the fitted ellipse. Because the actual twin plane is generally inclined to the scanning surface, the true twin thickness is estimated by multiplying the projected twin thickness by the cosine of the angle between the twinning plane, K1, and the scanning surface normal. The twin thickness at grain boundaries is estimated by measuring the length of the twins along the grain boundaries.

### Crystal plasticity model

The Fast Fourier Transform-based crystal plasticity models provide spatially resolved micromechanical fields in the individual crystals within polycrystals. The formulation provides an exact solution of the governing equations of equilibrium and compatibility, in such a way that the final (converged) equilibrated stress and compatible strain fields fulfill the constitutive relationship at every discrete material point. The original FFT formulation was developed to study the local and effective mechanical response of composite materials^53^. Later the FFT formulation was adapted for polycrystalline materials and permitted the study of the effective and local mechanical response associated with the heterogeneity in the spatial distribution of crystallographic grains^54^. In this work we use the elasto-visco plastic FFT formulation^55^ extended to account for the reorientation and twinning shear transformation in discrete regions within a crystal. In this model, deformation twinning is treated as a shear transformation process. Accordingly, the constitutive behavior of an elastic-visco-plastic material under an infinitesimal strain approximation with shear transformation becomes

2 $${{\boldsymbol{\sigma }}}^{t+{\rm{\Delta }}t}(x)={\bf{C}}(x):({{\boldsymbol{\varepsilon }}}^{t+{\rm{\Delta }}t}(x)-{{\boldsymbol{\varepsilon }}}^{p,t}(x)-{\dot{{\boldsymbol{\varepsilon }}}}^{{\rm{p}},{\rm{t}}+{\rm{\Delta }}t}(x){\rm{\Delta }}t-{{\boldsymbol{\varepsilon }}}^{{\rm{tr}},{\rm{t}}}(x)-{\rm{\Delta }}{{\boldsymbol{\varepsilon }}}^{{\rm{tr}},{\rm{t}}+{\rm{\Delta }}t}(x))$$

where **σ**(x) is the Cauchy stress tensor, **C**(x) is the elastic stiffness tensor, and **ε**(x), **ε**^**e**^(x), and **ε**^p^(x) are the total, elastic and plastic strain tensors. And **ε**^**tr**^ is the twinning shear transformation strain. During the build-up of the twinning transformation, successive shear increments are imposed in the twin domain and the system relaxed. The associated strain increments have the following relationship with the local twin variant at point *x*:

3 $${\rm{\Delta }}{{\boldsymbol{\varepsilon }}}^{tr}(x)={{\bf{m}}}^{tw}(x){\rm{\Delta }}{\gamma }^{tw}(x)$$

For material points lying outside the twin domain, ${\rm{\Delta }}{{\boldsymbol{\varepsilon }}}^{tr}\,$is zero. The tensor ${m}^{tw}=\frac{1}{2}({{\bf{b}}}^{{\rm{tw}}}\otimes {{\bf{n}}}^{{\rm{tw}}}+{{\bf{n}}}^{{\rm{tw}}}\otimes {{\bf{b}}}^{{\rm{tw}}})$ is the Schmid tensor associated with the twinning system, where **b**^tw^ a**n**d **n**^tw^ are unit vectors along the twinning direction and twin plane normal, respectively. The twinning transformation builds up in increments, until reaching the characteristic-twin shear, s^tw^.

4 $${\rm{\Delta }}{\gamma }^{tw}(x)=\frac{{s}^{{\rm{tw}}}}{{N}^{{\rm{twincr}}}}$$

The time increment Δt and the number of increments to achieve the twin transformation N^twincr^ are set sufficiently low and high, respectively, to ensure convergence.

## Data Availability

Data available on request from the authors: The data that support the findings of this study are available from the corresponding author upon reasonable request.
